# Phlebotomine sandfly (Diptera: Psychodidae) diversity and their *Leishmania* DNA in a hot spot of American Cutaneous Leishmaniasis human cases along the Brazilian border with Peru and Bolivia

**DOI:** 10.1590/0074-02760160054

**Published:** 2016-06-10

**Authors:** Carolina Bioni Garcia Teles, Ana Paula de Azevedo dos Santos, Rui Alves Freitas, Arley Faria José de Oliveira, Guilherme Maerschner Ogawa, Moreno Souza Rodrigues, Felipe Arley Costa Pessoa, Jansen Fernandes Medeiros, Luís Marcelo Aranha Camargo

**Affiliations:** 1 Fundação Oswaldo Cruz, Porto Velho, RO, Brasil; 2 Faculdade São Lucas, Porto Velho, RO, Brasil; 3 Universidade Federal de Rondônia, Porto Velho, RO, Brasil; 4 Instituto Nacional de Pesquisas da Amazônia, Manaus, AM, Brasil; 5 Fundação Oswaldo Cruz, Instituto Leônidas e Maria Deane, Manaus, AM, Brasil; 6 Universidade de São Paulo, Instituto de Ciências Biomédicas, Monte Negro, RO, Brasil

**Keywords:** sandflies, Leishmania, disease vectors, Assis Brasil Municipality, Acre, Brazil

## Abstract

In this study, we identified the phlebotomine sandfly vectors involved in the transmission of American Cutaneous Leishmaniasis (ACL) in Assis Brasil, Acre, Brazil, which is located on the Brazil-Peru-Bolivia frontier. The genotyping of *Leishmania* in phlebotomines was performed using polymerase chain reaction (PCR) and PCR-restriction fragment length polymorphism. A total of 6,850 sandflies comprising 67 species were captured by using CDC light traps in rural areas of the municipality. Three sandfly species were found in the state of Acre for the first time: *Lutzomyia georgii*, *Lu. complexa* and *Lu. evangelistai*. The predominant species was *Lu. auraensis*/*Lu. ruifreitasi* and *Lu. davisi* (total 59.27%). 32 of 368 pools were positive for the presence of *Leishmania* DNA (16 pools corresponding to *Lu. davisi*, and 16 corresponding to *Lu. auraensis/Lu. ruifreitasi*), with a minimal infection prevalence of 1.85% in *Lu. davisi* and 2.05% in *Lu. auraensis/Lu. ruifreitasi*. The *Leishmania* species found showed maximum identity with *L. (Viannia) guyanensis* and *L. (V.) braziliensis* in both phlebotomine species. Based on these results and similar scenarios previously described along the Brazil/Peru/Bolivia tri-border, the studied area must take into consideration the possibility of *Lu. davisi* and *Lu. auraensis/Lu. ruifreitasi* as probable vectors of ACL in this municipality.

The Amazon basin probably has the highest diversity of phlebotomine sandflies worldwide (Alves et al. 2012). Besides that, the Amazon basin has a high species endemicity (Ramos et al. 2014, Pereira Jr et al. 2015). In spite of this diversity, information about sandfly fauna in the state of Acre in the Brazilian Amazon, is still scarce (Teles et al. 2013b, Araujo-Pereira et al. 2014, Oliveira et al. 2015). These insects are relatively well studied in several places worldwide, due to their importance as vectors of protozoans, bacteria and viruses (WHO 2015).

In Brazil, seven *Leishmania* species are etiological agents of different clinical forms of American Cutaneous Leishmaniasis (ACL): *Leishmania (Viannia) braziliensis*, *L. (V.) guyanensis*, *L. (V.) lainsoni*, *L. (V.) naiffi*, *L. (V.) shawi* and *L. (V.) lindenbergi* representatives of the *L. (V.) braziliensis* complex; and *L. (L.) amazonensis* from the *L. mexicana* complex*.* All of these *Leishmania* species have been registered in the Brazilian Amazon (Lainson 2010).

In northern Brazil there is a wide range of geographically different places where sandflies can be found and also a variety of reservoirs and vector species from the subgenera *Nyssomyia*; *Psychodopygus*; *Lutzomyia* and *Trichophoromyia* (Castellón 2009, Feitosa et al. 2012). In the state of Pará, the species that stand out are *Lutzomyia complexa*, *Lu. wellcomei* and *Lu. longipalpis* (Aguiar & Medeiros 2003). In Amazonas the main vectors are *Lu. umbratilis*, *Lu. anduzei, Lu. flaviscutellata* and *Lu. wellcomei* (Aguiar & Medeiros 2003, Guerra et al. 2006).

The findings of different epidemiological profiles of leishmaniasis, together with records of outbreaks both in the wild as well as in peri-urban environments (WHO 2015), reinforces the hypothesis of the possible adaptation of vectors to changing environments and also the incorporation of domestic animals as reservoirs (Teles et al. 2013a, Araujo-Pereira et al. 2014).

The municipality of Assis Brasil, in the state of Acre, an area that borders Peru and Bolivia, located in the microregion of Brasiléia, is one of the most important ACL endemic areas of Brazil with high rates of the disease (43.6% of the ACL cases in Acre between 2011-2012 were concentrated in this municipality). In Assis Brasil, during the period from 2007-2012, the average detection rate was 98.2 cases/10,000 inhabitants, with 21.4% being mucosal form cases (SINAN 2014). In addition to the high rate of detection, this municipality has some important epidemiological characteristics like the permanence of a significant percentage (40%) of the population living in rural areas (IBGE 2014). Furthermore, after the opening of the Pacific Highway, migration across the triple border and the growth of tourism in the region increased (Cesário et al. 2011). This scenario may play an important role in the spread of ACL.

According to Teles et al. (2015), the most prevalent *Leishmania* species in humans from this municipality are *L. braziliensis* and *L. shawi*. Little is known about the vectors of this region, but some species suspected of or implicated in transmitting *Leishmania* spp. were reported in the state such as *Lutzomyia davisi, Lu*. *whitmani*, *Lu. antunesi*, *Lu. ubiquitalis* and *Lu. umbratilis* (Azevedo et al. 2008, Silva-Nunes et al. 2008). Sandfly studies in Acre are still scarce. Some entomological studies have been published to date, describing the sandfly fauna involving the cities of Cruzeiro do Sul, Feijó, Bujari, Xapuri, Rio Branco, Acre and Assis Brasil (Martins & Silva 1964, Arias & Freitas 1982, Azevedo et al. 2008, Silva-Nunes et al. 2008, Teles et al. 2013b, Araujo-Pereira et al. 2014, Oliveira et al. 2015).

Assis Brasil is one of the main epicenters of Amazonian ACL; however, there are information gaps on the epidemiological, clinical, vectorial and special aspects of *Leishmania* spp. circulating in this and various microregions of the state. The study reports for the first time, vectors and etiological agents from this border region in Acre.

## MATERIALS AND METHODS

**Fig. 1:**
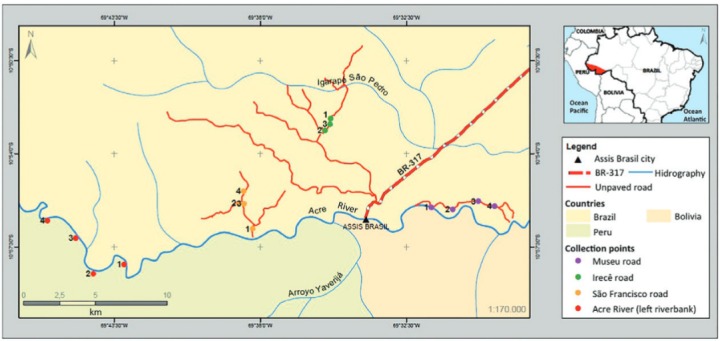
: Map of Brazil highlighting the state of Acre, location of the district headquarters of Assis Brasil municipality and points of sandfly capture (legends 1, 2, 3 and 4). Irecê road: point 1 (10º53’40.55”S/ 69º36’2.53”W); point 2 (10º53’3.35”S/ 69º35’25.95”W); point 3 (10º52’46.11”S/ 69º35’12.81”W). São Francisco road: point 1 (10º56’49.5”S/ 69º38’17.4”W); point 2 (10º55’49.40”S/ 69º38’49.96”W); point 3 (10º55’52.4”S/ 69º38’38.6”W); point 4 (10º55’22.6”S/ 69º38’36.0”W). Museu road: point 1 (10º56’0,95”S/ 69º31’33,54”W); point 2 (10º56’4.49”S/ 69º30’45.71”W); point 3 (10º55’46.97”S/ 69º29’47.54”W); point 4 (10º55’58.30”S/ 69º29’11.07”W). Riparian Forest: point 1 (10º58’09.4”S/ 69º43’07.8”W); point 2 (10º56’4.49”S/ 69º30’45.71”W); point 3 (10º55’46.97”S/ 69º29’47.54”W); point 4 (10º55’58.30”S/ 69º29’11.07”W).

*Study area* - The municipality of Assis Brasil (Fig. 1) occupies an area of 4.974 km^2^ in the state of Acre, Brazil, in the mesoregion of the Acre valley, Brasiléia microregion (10º56’29”S and 69º34’01”W). This area of Brazil borders Bolivia and Peru (Acre-Brazil, Madre de Dios-Peru and Pando-Bolivia). The Acre River marks the border between Assis Brasil and Bolivia. A population of 6,610 inhabitants occupies the area. The study area has a landscape formed by a mosaic of indigenous lands, extractive reserves, riverine communities, and small and large settlements. The local economy is focused on rubber, Brazil nuts, wood, vegetable oils and wild fruit. There are some small crop and livestock farms (Acre 2008).

The equatorial weather is hot and humid and the average annual temperature is 26.5ºC; the relative humidity is 80-90% all year round. There are two very distinct seasons: a wet season between November-April, and a dry season between May-October (IBGE 2014).

**Fig. 2:**
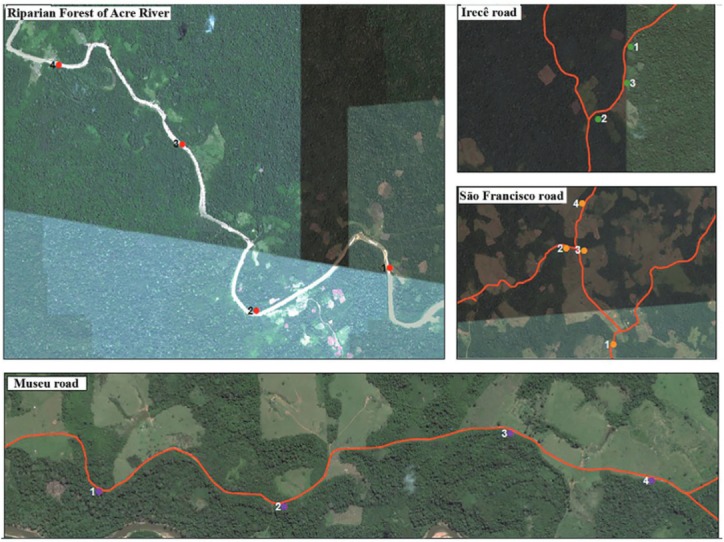
: landscape aspect of the sandfly collection areas, Assis Brasil Municipality, Acre state, Brazil. The satellite images were obtained through Google Earth (May 2010).

*Phlebotomine sandfly survey* - The phlebotomine sandfly surveys were done between August 2009-June 2010 with three or five CDC light traps/localities, placed approximately 150 cm above the ground with a distance of approximately 200 metres between them. Collections were carried out on five to eight consecutive nights from 18:00-06:00; a total of 780 captures were performed. During the dry season 26 visits were carried out totaling 448 traps installed in peridomiciliary environment (rural areas - Irecê road - capture August/2009; São Francisco road - capture September/2009; and Museu road - capture April and June/2010) according to the accessibility of roads and reports of recent cases of ACL (Figs 1-2). During the rainy season, the collections were held at four fixed points in riparian environments along the left bank of the river where 23 visits were carried out totaling 332 traps (capture November and December/2009 and February/2010).

The choice of the collection areas within each area of road were defined as follows: households with recent cases of ACL, within peridomicile areas possessing livestock, a fruitgarden and with primary or secondary fragments of forest. A transect of at least 800 metres was done; and the light traps were distributed throughout this transect. Points 1-3 of the riparian environment of the Acre River had native vegetation consisting of continuous rainforest and point 4 was in a peridomicile environment located in a fragment of forest near a riverside residence.

The sandflies collected were stored in 70% ethanol and then identified by the taxonomic key according to Young and Duncan (1994). The captured females were treated as follows: the head and the last three segments of the abdomen were clarified and mounted in the dorsal-ventral position in Berlese fluid; and the remainder of the body was stored in a microtube containing 70% ethanol for later molecular analysis. The males were slide mounted normally and then identified. Recently, *Lu.* (*Thrychophoromyia*) *ruifreitasi* was described after the identification from individuals collected for this study (Oliveira et al. 2015), and due to the fact that the females of *Lu. auraensis* and *Lu. ruifreitasi* are probably indistinguishable, we decided to temporarily name them *Lu. auraensis*/*Lu. ruifreitasi*.

*Detection of natural infection of sandflies by Leishmania sp.* - The collected females were assembled in groups (2-20 specimens per group) in micro test tubes. The single pool is composed with belonging the same sandfly species, date and place of capture. Total DNA was extracted from each pool based on the method adapted by Mukhopadhyay et al. (2000) and Paiva et al. (2007). After the extraction they were stored at 20ºC for polymerised chain reaction (PCR) in order to identify the genus *Leishmania*, complexes and *Leishmania* species.

*PCR targeted the mkDNA gene* - The PCR assay for the genus *Leishmania* (*mkDNA* gene PCR) was performed following Oliveira et al. (2005); the described primers 5’-GGG(GT)AGGGGCGTTCT(G/C)CGAA-3’ and 5’-(G/C)(G/C)(G/C)(A/T)CTAT(A/T)TTACACCAACCCC3’ target the conserved region of the kinetoplast DNA minicircles (mkDNA) (120 pb) among all the species of *Leishmania* sp.

*Multiplex PCR targeted the RNA mini-exon spliced leader (SL) gene* - In order to determine the three complexes of New World *L.* (*V.) braziliensis, L. (L.) mexicana* and *L. (L.) donovani*, the DNA from samples positive for *mkDNA* gene PCR were analysed through a second PCR, Multiplex PCR assays, with the specific primers for the SL RNA (mini-exon) gene (Harris et al. 1998). *SL RNA* Multiplex PCR uses simultaneously three specific primers for each complex: *L. braziliensis* complex LB-3C 5’-CGT(C/G)CCGAACCCCGTGTC-3’; *L. mexicana* complexLM-3A 5’-GCACCGCACCGG(A/G)CCAC-3’; *L. donovani* complex (LC-3L) 5’-GCCCGCG(C/T)GTCACCACCAT-3’ and one oligonucleotide primer which were each conserved in all *Leishmania* species LU-5A 5’-TTTATTATGCGAAACTTC-3’.

The size of the expected *SL RNA* Multiplex PCR product was 146 to 149 bp for the *L. braziliensis* complex, 218 to 240 bp for the *L. mexicana* complex, and 351 to 397 bp for the *L. donovani* complex.

*hsp70 gene PCR-restriction fragment length polymorphism (RFLP) and DNA sequencing* - In order to identify the *Leishmania* species from female sandflies, the hsp70 region was amplified (*hsp70* PCR) with primers hsp 70cF 5’-GGACGAGATCGAGCGCATG GT-3’ and hsp 70cR 5’-TCCTTCGACGCCTCCTGGTTG-3’ to amplify the 240 bp fragment (da Graça et al. 2012).

The final products amplified using *mkDNA* gene PCR*, SL RNA* Multiplex PCR and *hsp70* PCR were analysed on a 2% agarose gel stained with GelRed and examined under UV light.

For the RFLP analysis from the *hsp70* amplicons of 240 bp, the restriction enzymes HaeIII (Invitrogen®, USA) and BstUI (Bio Labs®, New England) were used in independent reactions according to the manufacturer’s instructions. The resulting products of the restriction digestion were analysed on 12% silver-stained polyacrylamide gels.

Negative controls such DNA from male sandflies was used for each PCR. The following DNA reference strains of the *Leishmania* collection from the Instituto Oswaldo Cruz (CLIOC) were used, namely: *L. (V.) braziliensis* (IOCL 566), *L. (V.) guyanensis* (IOCL 565), *L. (V.) lainsoni* (IOCL 1023), *L. (V.) naiffi* (IOCL 1365), *L. (V.) shawi* (IOCL 1545); *L. (L.) amazonensis* (IOCL 575) and *L. (L.) infantum* (IOCL 0579).

Sequencing of *Leishmania* DNA copies was carried out with a BigDye Terminator v3.1 Cycle Sequencing Kit (Applied Biosystems Brazil, São Paulo, Brazil) in an Applied Biosystems3130xl DNA sequencer from Genomic Company (São Paulo, Brazil). The *hsp70* genes of the *Leishmania* species were compared with the sequences obtained from analysis with reference sequences deposited in GenBank. Comparisons were done using BLAST program searches (Basic Local Alignment Search Tool, NCBI) (http://blast.ncbi.nlm.nih.gov).

*Pool screening* - Prevalence studies of infection in the sandflies by *Leishmania* spp. was assessed by estimating the prevalence of infection in positive pools using the Pool Screening Program (http://www.soph.uab.edu/bst/poolscreen) Version 2.0 (Katholi et al. 1995).

## RESULTS

A total of 6,850 phlebotomine were captured, 3,480 males (50.8%) and 3,370 females (49.2%), with a female/male ratio of 0.97. In all, 67 species were identified belonging to two genus, *Brumptomyia* (73 individuals, 1.08%) and *Lutzomyia* (6,777, 98.92%). The distribution and percentage of specimens of the genus *Lutzomyia* are presented in Table I, they belong to the following subgenera: *Trichophoromyia* (39.51%), *Psychodopygus* (27.34%), *Pressatia* (9.89%), *Nyssomyia* (8.58%). The other subgenera and species group made up less than 13.6%.

**TABLE I:** Abundance of phlebotomine sandflies collected with CDC light traps in Assis Brasil Municipality, state of Acre, Brazil, between August of 2009-June of 2010

Genus	Subgenera/Groups	Species	Environments						Total	(%)

			Peridomiciliary environment			Riparian environment				
	
			♀	♂	Total	♀	♂	Total		
*Brumptomyia*		*Brumptomyia avellari*	0	13	13	0	2	2	15	0,22
		*B. pentacantha*	3	41	44	2	1	3	47	0,7
		*B. sp.*	11	0	11	0	0	0	11	0,16
*Lutzomyia*	*Evandromyia*	*Lutzomyia monstruosa*	6	1	7	0	0	0	7	0,1
		*Lu. georgii* ^*a*^	7	0	7	0	0	0	7	0,1
		*Lu. tarapacaensis*	17	6	23	7	4	11	34	0,5
	*Lutzomyia*	*Lu. evangelistai* ^*a*^	4	0	4	2	0	2	6	0,09
		*Lu. flabellata*	0	6	6	0	0	0	6	0,09
		*Lu. sherlocki*	56	98	154	13	2	15	169	2,46
	*Micropygomyia*	*Lu. micropyga*	0	0	0	0	1	1	1	0,01
	*Nyssomyia*	*Lu. antunesi* ^*b*^	77	28	105	31	0	31	136	2
		*Lu. flaviscutellata* ^*b*^	1	6	7	2	4	6	13	0,19
		*Lu. reducta* ^*b*^	0	4	4	1	1	2	6	0,09
		*Lu. richardwardi*	10	0	10	35	1	36	46	0,67
		*Lu. shawi*	87	53	140	43	1	44	184	2,7
		*Lu. umbratilis* ^*b*^	1	0	1	0	0	0	1	0,01
		*Lu. whitmani* ^*b*^	67	56	123	6	0	6	129	1,89
		*Lu. yuilli yuilli*	51	17	68	0	0	0	68	1
		*Lu. christenseni*	0	2	2	0	0	0	2	0,03
	*Pintomyia*	*Lu. calcarata*	1	1	2	0	6	6	8	0,12
	*Pressatia*	*Lu. choti*	127	35	162	131	380	511	673	9,83
		*Lu. triacantha*	1	3	4	0	0	0	4	0,06
	*Psathyromyia*	*Lu. abonnenci*	0	1	1	0	0	0	1	0,01
		*Lu. campbelli*	1	1	2	0	0	0	2	0,03
		*Lu. dendrophyla*	12	77	89	2	0	2	91	1,33
		*Lu. lutziana*	8	30	38	0	2	2	40	0,58
		*Lu. punctigeniculata*	0	1	1	0	0	0	1	0,01
		*Lu. shannoni*	5	6	11	0	0	0	11	0,16
	*Psychodopygus*	*Lu. amazonensis*	11	9	20	5	0	5	25	0,36
		*Lu. ayrozai* ^*b*^	2	0	2	2	0	2	4	0,06
		*Lu. bispinosa*	1	0	1	1	0	1	2	0,03
		*Lu. carrerai*	20	7	27	15	1	16	43	0,63
		*Lu. davisi* ^*b*^	317	130	447	729	267	996	1443	21,07
		*Lu. geniculata*	23	2	25	65	22	87	112	1,64
		*Lu. hirsuta hirsuta* ^*b*^	23	20	43	36	11	47	90	1,31
		*Lu. llanosmartinsi*	0	2	2	0	2	2	4	0,06
		*Lu. lainsoni*	1	0	1	5	1	6	7	0,1
		*Lu. paraensis* ^*b*^	3	1	4	1	0	1	5	0,07
		*Lu. yucumensis*	3	0	3	71	62	133	136	2
		*Lu. complexa* ^*a,b*^	0	0	0	1	0	1	1	0,01
	*Sciopemyia*	*Lu. preclara*	3	1	4	6	11	17	21	0,3
		*Lu. servulolimai*	12	1	13	4	0	4	17	0,25
		*Lu. sordellii*	1	2	3	0	3	3	6	0,08
*Lutzomyia*	*Trichophoromyia*	*Lu. auraensis /Lu. ruifreitasi* ^b**^	232	229	461	665	1490	2155	2616	38,2
		*Lu. melloi*	0	0	0	1	0	1	1	0,01
		*Lu. ubiquitalis* ^*b*^	33	51	84	4	0	4	88	1,3
	*Viannamyia*	*Lu. furcata* ^*b*^	6	2	8	0	0	0	8	0,11
	Group *Aragaoi*	*Lu. abunaensis*	1	6	7	0	1	1	8	0,11
		*Lu. aragaoi*	31	66	97	14	41	55	152	2,22
		*Lu. brasiliensis*	0	3	3	0	0	0	3	0,04
	Group *Dreisbachi*	*Lu. dreisbachi*	3	1	4	0	0	0	4	0,06
	Group *Migonei*	*Lu. andersoni*	5	6	11	0	0	0	11	0,16
		*Lu. bacula*	1	4	5	0	0	0	5	0,07
		*Lu. migonei* ^*b*^	1	1	2	0	1	1	3	0,04
		*Lu. sallesi*	0	0	0	1	1	2	2	0,03
		*Lu. termitophila*	9	6	15	0	0	0	15	0,22
		*Lu. walkeri*	0	2	2	3	0	3	5	0,07
		*Lu. williamsi*	2	1	3	0	0	0	3	0,04
	Group *Oswaldoi*	*Lu. longipennis*	3	2	5	0	2	2	7	0,1
		*Lu. peresi*	0	1	1	0	0	0	1	0,01
		Lu. villelai*	1	0	1	0	0	0	1	0,01
	Group *Saulensis*	*Lu. saulensis*	45	12	57	3	1	4	61	0,89
		*Lu. wilsoni*	46	11	57	1	0	1	58	0,85
	Group *Verrucarum*	*Lu. nevesi*	18	22	40	34	10	44	84	1,2
		*Lu. serrana*	17	57	74	1	2	3	77	1,12
		*Lu. naiffi*	0	2	2	0	0	0	2	0,03
		Total	1427	1146	2573	1943	2334	4277	6850	100

*a*: new occurrence to Acre state; *b*: species are incriminated/suspected vectors (Rangel & Lainson 2009); b**: species are suspected vectors according to these study’s findings; *: taxon considers a synonym of *Lu. trinidadensis* by Young and Duncan (1994).

Among the 67 species, three were reported in the state of Acre for the first time: *Lu. georgii*, *Lu. complexa* and *Lu. evangelistai*. The most abundant taxa were *Lu. auraensis*/*Lu. ruifreitasi* (38.2%), *Lu. davisi* (21.07 %), *Lu. choti* (9.83%) and *Lu. shawi* (2.7%), which represent 71.8% of the total. The less abundant species were: *Lu. micropyga*, *Lu. umbratilis*, *Lu. abonnenci*, *Lu. punctigeniculata*, *Lu. complexa*, *Lu. melloi*, *Lu. peresi* and *Lu. villelai* (synonymy of *Lu. trinidadensis* by Young & Duncan 1994), representing 0.08% (Table I).

In the peridomiciliary environment 2,573 sandflies were collected, distributed among 63 species. The most abundant were *Lu. auraensis/Lu. ruifreitasi* (17.91%), *Lu. davisi* (17.37%), *Lu. choti* (6.3%), *Lu. sherlocki* (5.98%), *Lu. shawi* (5.44%), *Lu. whitmani* (4.78%) and 56 other species that occurred in small numbers for a total of 42.22%.

In the riparian forest environments, 4,277 sandflies were collected and distributed among 44 species. The most abundant were *Lu. auraensis/Lu. ruifreitasi* (50.38%), *Lu. davisi* (23.28%), *Lu. choti* (11.94%), *Lu. yucumensis* (3.1%), *Lu. geniculata* (2.03%), *Lu. aragaoi* (1.28%) and 38 other species amounting to 7.99%.

12 sandfly species are incriminated/suspected vectors of ACL (Rangel & Lainson 2009): *Lu. davisi, Lu. antunesi* and *Lu. whitmani* being the most abundant species, whereas the less abundant were *Lu. flaviscutellata*, *Lu. reducta*, *Lu. umbratilis*, *Lu. ayrozai*, *Lu. hirsuta hirsuta*; *Lu. paraensis*, *Lu. furcata*, *Lu. ubiquitalis*, *Lu. migonei* and *Lu. complexa* (Table I).

**Fig. 3:**
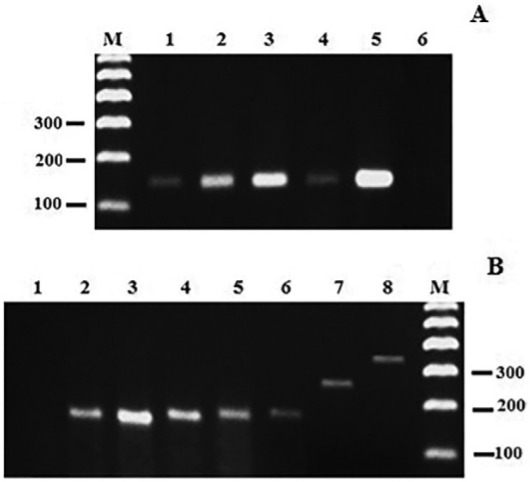
: *mkDNA* gene polymerase chain reaction (PCR) products and *SL RNA* Multiplex PCR resulting from sandfly pools. 2% agarose gel stained with GelRed. (A) *mkDNA* gene PCR. Lines 1-3: samples from *Lutzomyia davisi* pools; Line 4: *Lu. auraensis/Lu. ruifreitasi* pool; Line 5: *Leishmania (Leishmania) amazonensis* reference strain and Line 6: males of *Lutzomyia* sp. (B) Multiplex PCR for the *Leishmania* complex. Line 1: males of *Lutzomyia* sp.; Lines 2, 5: samples from *Lu. davisi* pools; Lines 3, 4: samples from *Lu. auraensis/Lu. ruifreitasi* pools; Lines 6-8 *Leishmania* spp. reference strains; Line 6: *L. (Viannia) braziliensis*; 7: *L. (L.) amazonensis*; 8: *L. (L.) infantum chagasi.* M: 100 bp size marker (Invitrogen).

A total of 3,218 (95.48%) females were grouped into 368 pools and 32 (8.7%) were positive for the *mkDNA* gene PCR specific for the *Leishmania* genus (16 pools corresponding to *Lu. davisi* and 16 to *Lu. auraensi*s/*Lu. ruifreitasi*) (Table II). The minimal infection prevalence for *Lu. davisi* was 1.85% (95% CI = 1.08% - 2.99%, n = 89 pools with 73 negatives) while *Lu. auraensis/Lu. ruifreitasi* was 2.05% (95% CI = 1.13% - 3.36%, n = 58 pools with 42 negatives). The Multiplex PCR technique identified all samples as related to the *L. braziliensis* complex (Fig. 3).

**TABLE II:** Distribution of positive sandfly pools for *mkDNA* gene polymerase chain reaction (PCR) according to species and collection environment, in Assis Brasil, AC

	Positive pools/Environment										*hsp70* PCR-restriction fragment length polymorphism

	Museu road				Forest Riparian						
Species (specimes - total pool)	P1	P2	P3	P4	P1	P2	P3	P4	Total	(%)	(nº pool*s*) *Leishmania* identification
*Lutzomyia auraensis/Lu. ruifreitasi* (1026-58)	3	1	-	-	9	1	-	2	16	50	(1) *L. (Viannia) braziliensis*
											(3) *L. (V.) guyanensis*
											(4) *L. (V.) guyanensis*/ *L. (V.) braziliensis*
*Lu. davisi* (853-89)	-	-	-	-	-	3	1	12	16	50	(1) *L. (V.) guyanensis*/ *L. (V.) braziliensis*
											(6) *L. (V.) guyanensis*
Total	3	1	0	0	9	4	1	14	32	100	

P: points of phlebotomine collection.

Most of the positive pools were found in riparian environments of the Acre River (28 pools - 87.0% covering all points), of which 14 pools (44.0%) were collected near riverside houses (point 4 of the riparian environment). Another four positive pools (13.0%) were collected at points 1 and 2 along Museu road (Table II).

**Fig. 4:**
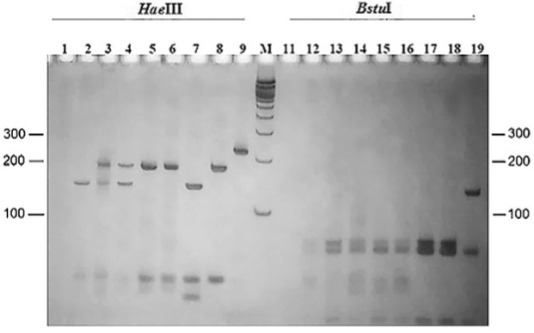
: polymerase chain reaction-restriction fragment length polymorphism (PCR-RFLP) for identification of *Leishmania* species in sandflies. The *hsp70* PCR products (234 bp) amplified within pools were digested by the enzymes *Hae*III and *Bstu*I. 12% silver-stained polyacrylamide gel. Lines 1, 11: Negative control of male *Lutzomyia* spp.; Lines 2, 12: *Lu. auraensis/Lu. ruifreitasi* pool I; Lines 3, 13: *Lu. davisi* pool II; Lines 4, 14: *Lu. auraensis/Lu. ruifreitasi* pool III; Lines 5, 15: *Lu. auraensis/Lu. ruifreitasi* pool IV; Lines 6, 16: *Lu. davisi* pool V; Lines 7, 17: positive control of *L. (Viannia) braziliensis*; Lines 8, 18: positive control of *L. (V.) guyanensis*; Lines 9, 19: positive control of *L. (L.) amazonensis*. M: 100 bp size marker (Invitrogen).

In this study, the *hsp70* PCR-RFLP analysis confirmed the *Leishmania* species of 15 phlebotomine pools (Table II), with two *Leishmania* species identified with restriction patterns equal to *L. (V.) guyanensis* (approximately *Hae*III 190 bp/*Bstu*I 50 bp) and *L. (V.) braziliensis* (*Hae*III 160 bp/*Bstu*I 50 bp) (Fig. 4)*.* Eight of the 15 *hsp70* PCR-RFLP from the positive pools were composed of *Lu. auraensis/Lu. ruifreitasi: L. (V.) guyanensis* (three pools), *L. (V.) braziliensis* (one) and *L. (V.) braziliensis/L. (V.) guyanensis* (four). Seven additional positive pools were composed of *Lu. davisi*: *L. (V.) guyanensis* (six) and *L. (V.) guyanensis/L. (V.) braziliensis* (one). The others sandfly samples did not yield any amplification product for *hsp70* PCR and it could not indicate the *Leishmania* species. However, all samples were positive for the *L. braziliensis* complex thought of Multiplex PCR.

Five samples with higher amplification yield by *hsp70* PCR were confirmed by genetic sequencing. Of these, three pools corresponding to *Lu. davisi* and two *Lu. auraensis/Lu. ruifreitasi* confirmed the presence and similarity (95-100%) of *L. (V.) guyanensis* deposited in GenBank under code number GU368213.1.

## DISCUSSION

Of the 67 captured species, three were recorded for the first time in Acre: *Lu. georgii*, *Lu. complexa* and *Lu. evangelistai*. The first species mentioned is considered endemic in northern Brazil. According to past records and this study, there are 92 known species for the state of Acre which corresponds to 34.6% of the registered species in Brazil (Aguiar & Medeiros 2003, Teles et al. 2013b, Galati 2014, Oliveira et al. 2015).

The number of species found in Assis Brasil was higher than that reported by other authors in Acre: Martins and Silva (1964) recorded 30 species of sandflies with a predominance of *Lu. nevesi* in Rio Branco and Arias and Freitas (1982), in a study carried out in the cities of Cruzeiro do Sul, Feijó and Rio Branco, identified 50 species, the most abundant being *Lu. auraensis*. Azevedo et al. (2008) identified 52 species in the municipalities of Bujari, Xapuri and Rio Branco, 67% of them being *Lu. auraensis*, *Lu. antunesi*, *Lu. whitmani* and *Lu. davisi*. Silva-Nunes et al. (2008) in the municipality of Acrelândia observed that *Lu. antunesi* (45%) and *Lu. whitmani* (15%) were the most abundant of the 40 collected specimens. Araujo-Pereira et al. (2014) in Rio Branco captured 23 species of sandflies with 53.3% *Lu. auraensis* and 18.8% *Lu. whitmani*. These studies indicated that knowledge of sandfly fauna is still scarce compared to other Brazilian states of the Amazon Basin.

The abundance the subgenera of *Trichophoromyia*, *Psychodopygus*, *Pressatia* and *Nyssomyia* found in this study is consistent with these studies performed in Acre (Arias & Freitas 1982, Azevedo et al. 2008, Silva-Nunes et al. 2008), with a high abundance of *Lu. (Trichophoromyia*) *auraensis*, *Lu.* (*Psychodopygus*) *davisi* and *Lu.* (*Pressatia*) *choti*. These three species amounted to 85.62% of total individuals caught in the riparian environment and 41.58% in the rural area. This profile of few dominant species and many species with few individuals has been reported in sandfly fauna studies in the north Brazil (Azevedo et al. 2008, Feitosa et al. 2012, Ramos et al. 2014).

We highlighted the findings of *Lu. davisi* and *Lu. auraensis/Lu. ruifreitasi* containing DNA tracks of *Leishmania* from the *L. braziliensis* complex. The minimal infection prevalence observed for these two species were: *Lu. davisi* with 1.85% and *Lu. auraensis/Lu. ruifreitasi* with 2.05%. To date, only Valdivia et al. (2012) observed *Lu. davisi* and *Lu. auraensis* through positive qPCR diagnosis in Madre de Dios department, Peru (0.9% and 0.6% respectively), associated with the transmission of *L. (V.) lainsoni* and *L. (V.) braziliensis*. Other studies confirming infection by *Leishmania* spp. in *Lu. davisi* were based on a conventional method of identifying flagellates using monoclonal antibodies (Grimaldi Jr et al. 1991, Gil et al. 2003, Souza et al. 2010).

This is the first report of infection of *Lu. auraensis*/*Lu. ruifreitasi* by *L. (V.) guyanensis* and *L. (V.) braziliensis* in Brazil. Teles et al. (2015) found both *Leishmania* species in tissue biopsies from local patients with ACL. *Lutzomyia auraensis* is present in northern Brazil with low abundance in Amazonas and Rondônia (Castellón et al. 1994, Gil et al. 2003, Teles et al. 2013a), and high abundance in Acre (Arias & Freitas 1982, Azevedo et al. 2008, Araujo-Pereira et al. 2014). Araujo-Pereira et al. (2014) reported the abundance of *Lu. auraensis* in peri-urban (city parks) and peridomiciliary environments in Rio Branco. However, no studies incriminating this sandfly as a vector have been conducted in Brazil, and its epidemiological role has not been well investigated so far. This species has also demonstrated a high abundance and anthropophilic behavior in Peru (Arístides 1999, Quispe et al. 2008, Valdivia et al. 2012). This evidence combined with molecular detection of *Leishmania* from the *L. braziliensis* complex bring to light the question of the role of *Lu. auraensis/Lu. ruifreitasi* as a suspect in the transmission of ACL.

The finding of *Lu. davisi* infected by *Leishmania* reinforces the importance of this species in the region, since it is considered a possible vector of *L. (V.) braziliensis* and *L. (V.) naiffi* in Rondônia (Grimaldi Jr et al. 1991, Gil et al. 2003) and in Pará (Souza et al. 2010). Despite its wild habitat, *Lu. davisi* has been found in peridomestic locations with the occurrence of ACL (Gomes et al. 2013, Teles et al. 2013a). In this study, most of the positive pools of *Lu. davisi* containing *Leishmania* DNA were collected near riparian forest. *Lu. davisi* is anthropophilic and the relationship between the density of this species, its possible adaptation to anthropogenic environments, together with the finding of natural infection of *L. (V.) braziliensis* and *L. (V.) guyanensis* in this study, reinforce the hypothesis of its involvement in enzootic and zoonotic cycles in the Amazon Region (Grimaldi Jr et al. 1991, Gil et al. 2003, Souza et al. 2010, Valdivia et al. 2012).

The local circulation of *L. (V.) braziliensis* and *L. (V.) guyanensis* has important epidemiological considerations since these species are endemic and responsible for cases of mucosal leishmaniasis in the Amazon Region (Guerra et al. 2006, Teles et al. 2015). *Leishmania (V.) braziliensis* has a higher diversity of potential vectors incriminated in its transmission in the literature (i.e. *Lu. choti*, *Lu. yucumensis*, and *Lu. carrerai carrerai* noted in this study); however, the principal described vector of *L. (V.) guyanensis* is *Lu. umbratilis* whose density was low in this study. The identification by *hsp70* PCR-RFLP of *L. (V.) guyanensis* in 14 pools (14/15) at different collection points and the high density of *Lu. auraensis*/*Lu. ruifreitasi* do not exclude the possibility that these sandflies participates in the transmission cycle of this parasite in the border area. In this sense, further studies are needed to assess the vector capacity such as through dissection of the sandflies digestive tube for detection of the parasite; an anthropophilic behavior study and one on blood feeding in other vertebrate hosts (Killick-Kendrick 1990).

The record of clinical and epidemiological samples in Assis Brasil from other *Leishmania* species: *L. (V.) shawi* and *L. (L.) amazonensis* (Teles et al. 2015) as well as *L. (V.) lainsoni* and *L. (V.) naiffi* in humans from the microregion of Rio Branco (Tojal da Silva et al. 2006) confirm the epidemiological importance of potential vectors found in the fauna reported in this study, such as: *Lu. paraensis*, *Lu. ayrozai* and *Lu. hirsuta hirsuta*, which have been found naturally infected by *L. (V.) naiffi* (Rangel & Lainson 2009); *Lu. flaviscutellata* which is considered the main vector of *L. (L.) amazonensis*, an etiological agent of the diffuse form of cutaneous leishmaniasis (Lainson et al. 1994, Lainson 2010); and *Lu. ubiquitalis*, a potential vector of *L. (V.) lainsoni* in Pará (Silveira et al. 1991, Lainson 2010).

Another significant fact in this study is the prevalence of the species *Lu. whitmani*, *Lu. antunesi* and *Lu. ubiquitalis* in the peridomiciliary environment, while in the riparian environment these species were collected in smaller quantities. These species adapt to changing environments (Rebêlo et al. 1999, Araujo-Pereira et al. 2014) and are important in the transmission of *L. (V.) braziliensis* (Queiroz et al. 1994), *L. (V.) guyanensis* (Freitas et al. 2002) and *L. (V.) shawi* (Lainson et al. 1994). The correlation of such species density and their presence in anthropogenic environments as observed by Azevedo et al. (2008), Silva-Nunes et al. (2008) and Araujo-Pereira et al. (2014) in Acre indicate *Lu. whitmani*, along with *Lu. antunesi*, as suspicious insect vector species in the region.

*Lu. antunesi* has been associated with wild and peridomestic environments (Rebêlo et al. 1999, Silveira et al. 2002, Ramos et al. 2014) and occurs in Peru and Bolívia (Galati 2014). This species was associated with visceral leishmaniasis outbreaks and the transmission of *L. (V.) lindenbergi* in Pará (Ryan et al. 1984, Silveira et al. 2002). Recently, Trujillo et al. (2013) highlighted the epidemiological importance of *Lu. antunesi* in the transmission of *Leishmania* spp. in an endemic area in Colombia for its high infection rate (1.6%), abundance and adaptation in intra and peridomiciliary environments.

Regarding *Lu. ubiquitalis*, this species has been related to a variety of habitats including areas surrounding homes and forested areas in the Amazon (Ramos et al. 2014). Despite its low anthropophily in natural environments, *Lu. ubiquitalis* is the first representative of the subgenus *Trichophoromyia* implicated as a potential vector of *L. (V.) lainsoni* in Pará (Silveira et al. 1991, Lainson 2010).

The record of *Lu. shawi* in this study (frequency of 2.7%) deserves attention when considering the epidemiological surveillance of leishmaniasis around the border. This species has recently been implicated in the dissemination of *Leishmania* in Bolivia supported by evidence of its anthropophilic character, its occurrence and abundance in endemic areas of ACL and in peridomiciliary environments, as well as its proven infection by *L. (V.) braziliensis* and *L. (V.) guyanensis* (Garcia et al. 2007, Bustamante et al. 2012).

Thus, the data from this study demonstrate the great diversity of sandflies species with potential involvement in the leishmaniasis transmission cycle in Assis Brasil. In addition, the abundance of *Lu. davisi* and *Lu. auraensis/Lu. ruifreitasi* with several positive pools for the *L. braziliensis* complex increases the data about vector suspects in the north Brazil and suggests the need for new studies proving such species as vectors in the ACL cycle.
